# Co-continuous structural effect of size-controlled macro-porous glass membrane on extracellular vesicle collection for the analysis of miRNA

**DOI:** 10.1038/s41598-021-87986-2

**Published:** 2021-04-21

**Authors:** Hiroshi Yukawa, Shuji Yamazaki, Keita Aoki, Kengo Muto, Naoto Kihara, Kazuhide Sato, Daisuke Onoshima, Takahiro Ochiya, Yasuhito Tanaka, Yoshinobu Baba

**Affiliations:** 1 Institute of Nano-Life-Systems, Institutes of Innovation for Future Society, Nagoya University, Furo-cho, Chikusa-ku, Nagoya, 464-8603 Japan; 2 Department of Biomolecular Engineering, Graduate School of Engineering, Nagoya University, Furo-cho, Chikusa-ku, Nagoya, 464-8603 Japan; 3 Institute of Quantum Life Science, National Institutes for Quantum and Radiological Science and Technology, Anagawa 4-9-1, Inage-ku, Chiba, 263-8555 Japan; 4 Nagoya University Institute for Advanced Research, Advanced Analytical and Diagnostic Imaging Center (AADIC)/Medical Engineering Unit (MEU), B3 Unit, Nagoya University, Tsurumai-cho 65, Showa-ku, Nagoya, 466-8550 Japan; 5 AGC Inc., 1-5-1, Marunouchi, Chiyoda-ku, Tokyo, 100-8405 Japan; 6 Nagoya University Institute for Advanced Research, S-YLC, Furo-cho, Chikusa-ku, Nagoya, 464-8603 Japan; 7 Department of Molecular and Cellular Medicine, Institute of Medical Science, Tokyo Medical University, Shinjuku, Shinjuku-ku, Tokyo, 160-8402 Japan; 8 Department of Gastroenterology and Hepatology, Faculty of Life Sciences, Kumamoto University, Honjo 1-1-1, Chuo-ku, Kumamoto, 860-8556 Japan; 9 College of Pharmacy, Kaohsiung Medical University, Shin-Chuan 1st Rd., Kaohsiung, 807 Taiwan, ROC

**Keywords:** Nanobiotechnology, Isolation, separation and purification

## Abstract

Recent studies have shown that extracellular vesicles (EVs) can be utilized as appropriate and highly specific biomarkers in liquid biopsy for the diagnosis and prognosis of serious illness. However, there are few methods that can collect and isolate miRNA in EVs simply, quickly and efficiently using general equipment such as a normal centrifuge. In this paper, we developed an advanced glass membrane column (AGC) device incorporating a size-controlled macro-porous glass (MPG) membrane with a co-continuous structure to overcome the limitations of conventional EV collection and miRNA extraction from the EVs. The size of macro-pores in the MPG membrane could be accurately controlled by changing the heating temperature and time on the basis of spinodal decomposition of B_2_O_3_, Na_2_O, and SiO_2_ in phase separation. The AGC device with an MPG membrane could collect the EVs simply and quickly (< 10 min) from cell culture supernatant, serum and urine. This AGC device could extract miRNA from the EVs captured in the MPG membrane with high efficiency when combined with a miRNA extraction solution. We suggest that the AGC device with an MPG membrane can be useful for the diagnosis and prognosis of serious illness using of EVs in various kinds of body fluids.

## Introduction

Liquid biopsy has received a great deal of attention because it enables us to diagnose serious illness simply and noninvasively compared with a traditional tissue biopsy via blood samples or other kinds of body fluids^1,2^. It is expected to apply for the super early diagnosis and prognosis (recurrence and metastasis) of tumor diseases, and to reduce the morbidity and mortality of cancer. However, effective and highly-specific biomarkers derived from body fluids are essential for the appropriate diagnosis and prognosis by liquid biopsy. Recent studies have shown that EVs can be utilized as appropriate and highly specific biomarkers in liquid biopsy for the diagnosis and prognosis of tumor diseases^3–7^.

EVs of 50–500 nm in diameter are abundant in many types of body fluids including serum, urine and saliva^8,9^. EVs containing miRNAs, DNA and proteins (metabolic products) are released after the fusion of multivesicular endosomes with plasma membrane, and have been proven to show the specific characteristics of origin cells^10–13^. In particular, it has been demonstrated that miRNA in tumor-derived EVs strongly influences the tumor microenvironment^14–17^. We have also confirmed that the miRNAs in EVs derived from hepatocellular carcinoma (HepG2) cells activate the angiogenesis of human umbilical vein endothelial cells (HUVECs)^18^. Thus, miRNAs in EVs have emerged as a useful source of biomarkers for the diagnosis, prognosis of serious illness and for treatment monitoring purpose.

The major established technical methods for collecting and isolating EVs include ultracentrifugation (UC), immune affinity approach (magnetic beads) and density-gradient separation^19^. UC is regarded as the gold standard for EV isolation, but this method is extremely time-consuming (> 8 h) and requires large sample volumes^20,21^. The immune affinity approach has been shown to be an effective method for the isolation of specific EV population; however, this method strongly relies on the antibody selectivity and specificity^8,22^. The density-gradient method improves the purity and recovery rate in comparison to UC, but makes it difficult to separate from large microvesicles due to their similar density.

Other methods such as size exclusive chromatography (SEC), the polymeric precipitation approach by using agents such as polyethylene glycol (PEG), and micro-nanofabrication devices also have been reported. SEC can separate EVs from proteins and some lipoproteins; however, the samples that include EVs are usually diluted in a certain volume of liquid^20^. The polymeric precipitation approach can wrap dozens or hundreds of EVs and these wraps are easily collected by low-speed centrifugation; however, it is limited by small sample volumes and suffers from high purity^23,24^. Micro-nanofabrication devices can effectively isolate EVs from small volumes of biofluids, and several microfluidic devices could also achieve the co-isolation and detection of EVs from clinical samples; however, they are limited to low throughput application and are specialized equipment with a method that has low versatility^9,11,20,25–27^.

In this study, we focused on the characterization of co-continuous structure in a size-controlled macro-porous glass (MPG) membrane for EV collection and the analysis of miRNA. It is generally accepted that inorganic porous glass has numerous features^28–31^. The pore size can be controlled sharply ranging from several nm to µm. The framework of the inorganic porous glass is composed of oxide, and thus has excellent heat resistance and is not affected by various types of organic solvents, acids, or decomposition by microorganisms. Inorganic porous glass has excellent mechanical strength, and thus shows dimensional stability in vessels. Moreover, modification of the surface can be achieved relatively simply.

We therefore developed an advanced glass membrane column (AGC) device incorporating a size-controlled macro-porous glass (MPG) membrane with a co-continuous structure to overcome the limitations of conventional EV collection and miRNA extraction from EVs. The MPG membrane was composed of silicon dioxide (SiO_2_) as a principal component of a structure, and a co-continuous structure was formed by spinodal decomposition. When combined with a miRNA extracting solution, our AGC column is a useful tool for the simple and quick collection of EVs and the extraction of miRNA from the EVs. To the best of our knowledge, this is the first study to apply the co-continuous structure formed by spinodal decomposition in an MPG membrane to EV collection and the analysis of miRNA.

## Results

### Fabrication of the AGC device

**Figure 1:**
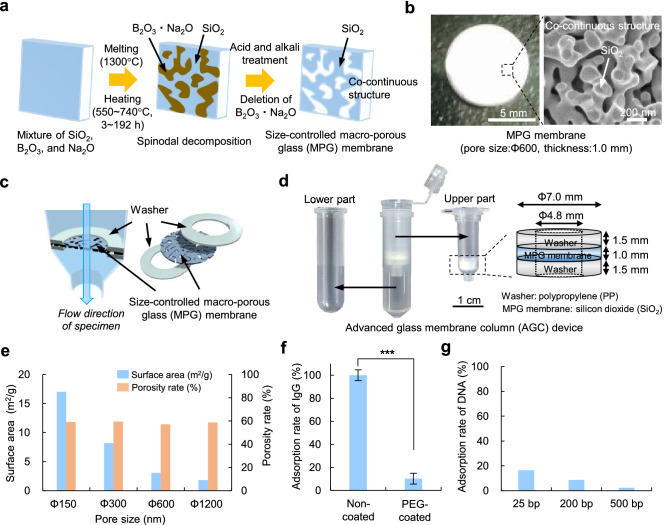
Fabrication of the AGC device. (**a**) The production process for producing a size-controlled macro-porous glass (MPG) membrane. (**b**) A photograph and SEM image of the MPG membrane. (**c**,**d**) An image (**c**) and photograph (**d**) of the advanced glass membrane column (AGC) device. The MPG membrane in the AGC device was sandwiched by washers. The AGC device was composed of two parts (upper part: EV collection, lower part: supernatant collection). (**e**) The surface area (m^2^/g) and porosity rate (%) of the membranes (outside diameter: 7.0 mm, thickness: 1.0 mm, pore size: Φ150, Φ300, Φ600, Φ1200 nm). (**f**) The adsorption rate (%) of IgG in the non-PEG coated and PEG coated MPG membranes. (**g**) The adsorption rate (%) of dsDNA (25 bp, 200 bp, 500 bp) in the PEG coated MPG membranes.

To collect the EVs from various types of samples, including cell culture medium and body fluids such as serum and urine, a size-controlled macro-porous glass (MPG) membrane was synthesized (Fig. 1a). The size of the macro-pores in the MPG membrane could be controlled by change the heating temperature and the time on based on the spinodal decomposition of B_2_O_3_, Na_2_O, and SiO_2_ in phase separation (Fig. S1). This method made it possible to control the pore size of the macro-porous structure in MPG membrane from 10 to 1200 nm (Fig. S2). A photograph of the MPG membrane (pore size: 600 nm, thickness: 1.0 mm) and an SEM image of the co-continuous structure in the MPG membrane are shown in Fig. 1b.

To apply the MPG membrane to the collection of EVs conveniently, efficiently, and in a short time, an advanced glass membrane column (AGC) device was developed, in which the MPG membrane was sandwiched by washers (Fig. 1c,d). The surface area and porosity of each MPG membrane (outside diameter: 7.0 mm, thickness: 1.0 mm, pore size: Φ150, Φ300, Φ600, Φ1200 nm) are shown in Fig. 1e. When the porosity rate was fixed to approximately 60% in every MPG membrane, the pore size of the nano-pores changed. The surface area of the MPG membrane gradually decreased with the increase of the pore size due to this change (Fig. 1e, Fig. S3). To suppress the adsorption of proteins and DNA fragments included in samples to the surface of the MPG membrane, the MPG membrane was coated with PEG polymer. A 90% reduction in the adsorption of IgG was observed in the PEG-coated MPG membrane relative to the non-coated membrane (Fig. 1f). Moreover, an inhibition of the adsorption of bovine serum albumin (BSA) and human serum albumin (HSA) was observed in the PEG-coated MPG membrane (Fig. S4, Supporting Information). Similarly, more than 85% reductions in the adsorption of dsDNA (25 bp, 200 bp, 500 bp) were observed in the PEG-coated MPG membrane (Fig. 1g).

It has recently been reported that the miRNA extracted from EVs plays an important role in the diagnosis and prognosis (recurrence and metastasis) of serious illnesses including tumor disease^30–32^. Consequently, various methods for the collection and isolation of EVs have been developed. However, there are few methods that can simply, quickly and efficiently collect EVs and isolate miRNA using general equipment such as normal centrifuge. We developed an advanced glass membrane column (AGC) device with a size-controlled macro-porous glass (MPG) membrane to overcome these limitations of conventional EV collection and the extraction of miRNA from the EVs. The MPG membrane was composed of SiO_2_ and had a stable co-continuous structure formed by spinodal decomposition. The pore-size of the MPG membrane could be accurately controlled by the heating temperature and the time under the condition of 60% porosity. The PEG coating of the surface significantly inhibited the prehension and adhesion of IgG and DNA molecules to the MPG membrane.

### EV collection using the AGC device

**Figure 2:**
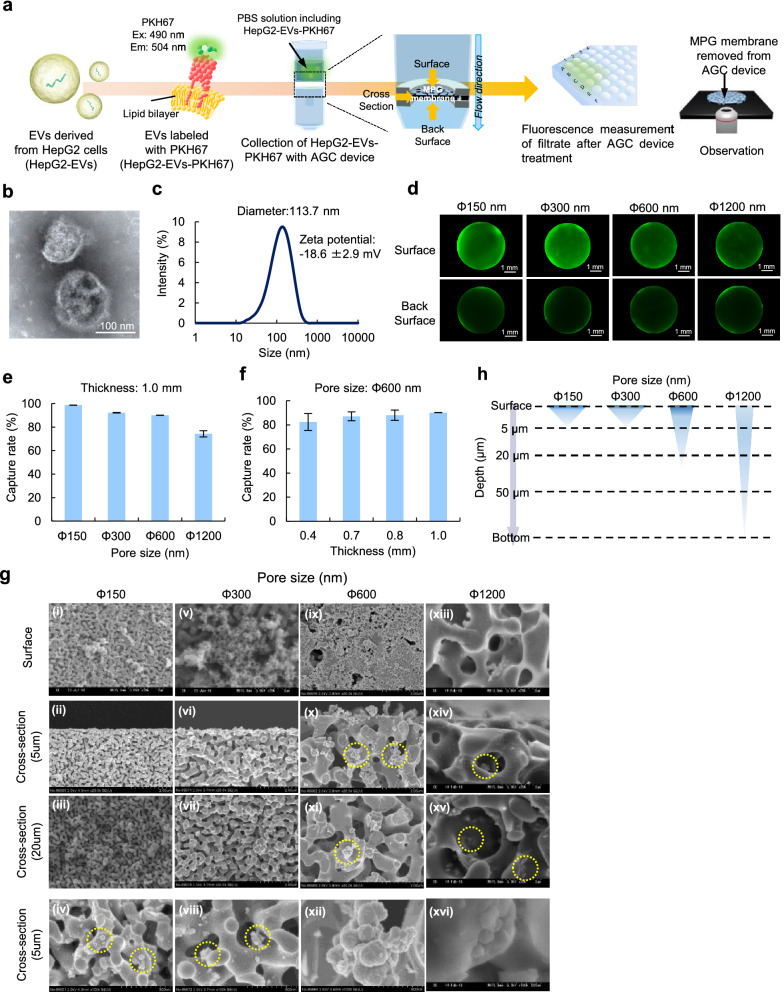
EV collection using the AGC device. (**a**) A schematic illustration of EV collection using the AGC device and the evaluation of the collection efficiency using a plate reader and fluorescence microscope. (**b**) A TEM image of EVs derived from HepG2 cells (HepG2-EVs). (**c**) The size and zeta potential distribution of HepG2-EVs. (**d**) A fluorescence image of an MPG membrane after the collection of HepG2-EVs labeled with PKH67 (green fluorescence) using the AGC device. (**e**,**f**) The capture rate (%) of HepG2-EVs by MPG membranes of different pore size (**e**) and thickness (**f**). (**g**) A SEM image of HepG2-EVs collected in MPG membranes with different pore sizes. (**h**) The schematic illustration demonstrating the depth at which HepG2-EVs could be observed in the MPG membrane with different pore sizes.

A conceptual diagram of a PEG coated MPG membrane for EV collection and a schematic diagram of the evaluation of capture rate (%) of EVs are shown in Fig. 2a. EVs in the cell culture medium in which HepG2 cells were incubated for four days were collected by ultracentrifugation. The EVs derived from HepG2 cells (HepG2-EVs) were observed using a transmission electron microscope (Fig. 2b). The average particle size was found to be 113.7 nm (range 20–300 nm), and the zeta potential of the EVs was − 18.6 ± 2.9 mV, as determined by a zeta-size analysis (Fig. 2c).

In order to check the collection of HepG2-EVs, the EVs were labeled with PKH67 agent with green fluorescence. Green fluorescence derived from HepG2-EVs labeled with PKH67 could be observed in all MPG membranes independent of the differences (Φ150 nm, Φ300 nm, Φ600 nm, Φ1200 nm and Φ3000 nm) in the pore size of the MPG membrane (thickness: 1.0 mm) (Fig. 2d). The capture rate was evaluated based on the fluorescence intensity of the PBS solution including HepG2-EVs labeled with PKH67 before and after AGC device treatment. The capture rate of HepG2-EVs was 98.8% at Φ150 nm, 92.2% at Φ300 nm, 90.1% at Φ600 nm, 74.4% at Φ1200 nm and 45.0% at Φ3000 nm (Fig. 2e, Fig. S5). More than 90% of the EVs could be collected in the MPG membrane of the AGC device, except for at the Φ1200 nm and Φ3000 nm condition.

To examine the influence of the thickness of the MPG membrane on the collection of EVs, MPG membranes (thickness: 0.4 mm, 0.7 mm, 0.8 mm, 1.0 mm, pore size: Φ600 nm) were prepared, and their EV capture rates were evaluated. The capture rate gradually increased with the thickness of the MPG membrane (Fig. 2f). HepG2-EVs collected in MPG membranes could be observed by SEM (Fig. 2g,h). HepG2-EVs was mainly observed on the surface or within a depth of 5 µm from the surface of the Φ150 nm and Φ300 nm MPG membranes. On the other hand, HepG2-EVs could be observed within a depth of 20 µm or more from the surface of the MPG membranes of greater than Φ600 nm. In addition, the time taken to collect EVs using the Φ600 nm MPG membrane was quite short in comparison to the Φ150 nm and Φ300 nm MPG membrane. Thus, we selected the Φ600 nm MPG membrane (thickness: 1 mm) for EV collection. The centrifugation speed of the AGC column with an MPG membrane (pore-size: Φ600 nm, thickness: 1 mm) did not affect the capture rate of HepG2-EVs (Fig. S6, Table S1).

Moreover, the ability of the AGC device with an MPG membrane (pore-size: Φ600 nm, thickness: 1 mm) to capture nanoparticles and other kind of EVs was checked. The capture rate of polystyrene beads (size: 50 nm and 100 nm) was approximately 100%. (Fig. S7). The capture rate of another kind of EVs collected from the cell culture supernatant of adipose tissue-derived stem cells (ASCs-EVs; size: 107.8 nm, zeta potential: − 11.0 ± 1.8 mV) was 93.3% (Fig. S8).

### Comparison of four EV collection methods

**Figure 3:**
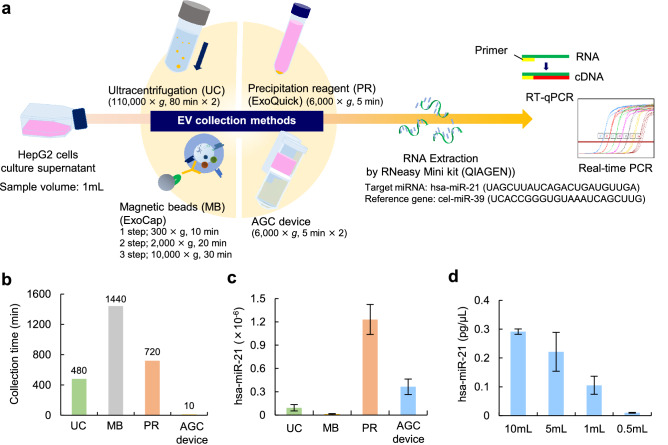
Comparison of four EV collection methods. (**a**) A schematic diagram of the real-time PCR analysis of has-miR-21 extracted from HepG2-EVs collected from cell culture supernatant using four EV collection methods (ultracentrifugation [UC], ExoCap [MB], ExoQuick [PR] and the AGC device). (**b**) The isolation time of HepG2-EVs in UC, MB, PR and the AGC device. (**c**) The detected level of hsa-miR-21 in UC, PR, MB and the AGC device. (**d**) The detected level of hsa-miR-21 in the AGC device with different volumes of cell culture supernatant.

A schematic diagram of the miRNA analysis of HepG2-EVs collected from cell culture supernatant is shown in Fig. 3a. The real-time PCR analysis of miRNA extracted from HepG2-EVs was performed to determine the quantity of miR-21, which is known as one of the representative mammal-related miRNAs. Three types of conventional EV collection methods (Ultracentrifugation [UC], ExoCap [MB] and ExoQuick [PR]) were also tested.

EVs were collected with each method from 1 mL of HepG2 cell culture supernatant and then the amount of hsa-miR-21 was measured. The time required to collect EVs using the AGC device (our method) was quite short in comparison to the other three methods. The isolation time using the AGC device was 10 min and was confirmed to be very shorter than the other three methods (Fig. 3b). In addition, the AGC device exhibited large amount of hsa-miR-21 in comparison to the UC and MB methods (Fig. 3c). To evaluate the effect of sample enrichment in the AGC device on the volume of collected miRNA, 1 mL, 5 mL, and 10 mL of cell culture supernatant were treated with the AGC device. The AGC device could filtrate to 500 µL of sample per filtration treatment. The increase in sample volume resulted in an increase in the amount of hsa-miR-21. The concentration effect of EVs could be confirmed in the AGC device (Fig. 3d).

### The miRNA array analysis of EVs collected from human serum

**Figure 4:**
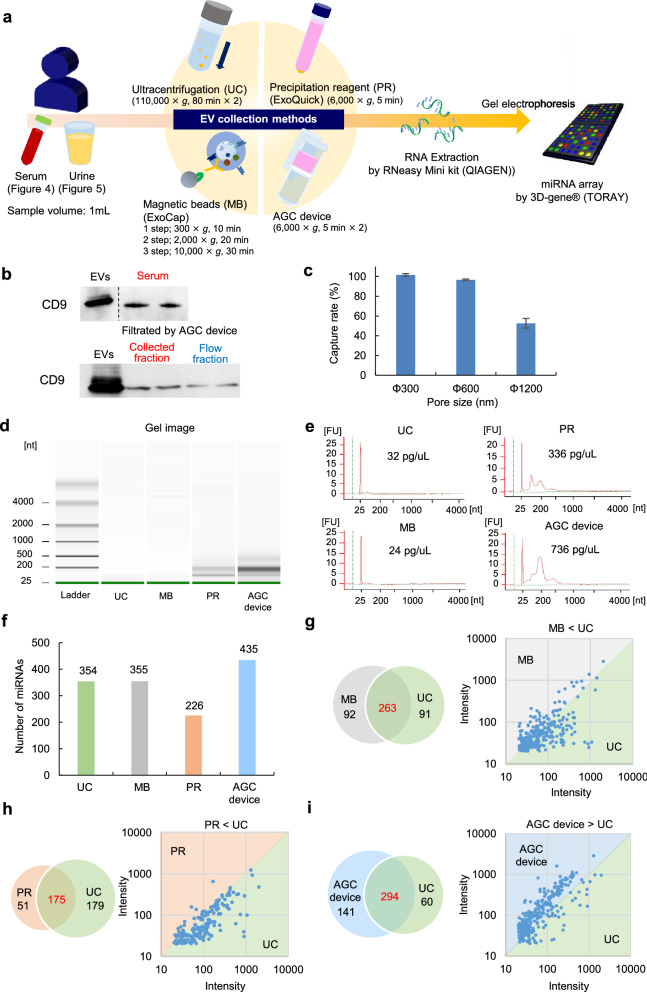
The miRNA array analysis of EVs collected from human serum. (**a**) A schematic diagram of the miRNA array analysis of HepG2-EVs collected from human serum and urine using four EV collection methods (UC, MB, PR, and the AGC device). (**b**) The western blotting of CD9 in serum (upper), and CD9 in the collected and flow fraction of serum treated by AGC device (lower). These blots were cropped from the same gel. The black dot line shows the delineation with dividing from different parts of the same gel. All information about full-length gels and blots was provided in Fig S9. (**c**) The capture rate (%) of EVs using AGC device calculated by CD9/CD63 ELISA kit. (**d**) A gel image of nucleotides [nt] of total RNA extracted from EVs collected from human serum using four EV collection methods (UC, MB, PR, and AGC device) measured by a 2100 Bioanalyzer. (**e**) The concentration of total RNA extracted from EVs collected from human serum using four EV collection methods (UC, MB, PR and the AGC device) measured by a 2100 Bioanalyzer. (**f**) The number of miRNAs extracted from EVs collected from human serum using four EV collection methods (UC, MB, PR, and the AGC device). (**g**–**i**) A venn diagram of the overlap of the number of detected miRNAs between UC (as the standard method) and the other three methods (MB [**g**], PR [**h**] and the AGC device [**i**]).

A schematic diagram of the miRNA array analysis of EVs collected from human serum is shown in Fig. 4a. CD9 (a commonly used as EV marker protein) was detected in the samples from 100 µL of serum. The collected fraction from EVs in serum, which was captured in AGC device, was confirmed to contain CD9 (Fig. 4b). In addition, in order to check the capture rate of EVs, the rate was analyzed using CD9/CD63 ELISA kit. The capture rate of EVs was about 100% at Φ300 nm, 96% at Φ600 nm, and 53% at Φ1200 nm as with Fig. 2e (Fig. 4c). These data suggested that more than 95% of the EVs could be collected in MPG membrane (pore-size: < Φ600 nm, thickness: 1 mm) of AGC device, and miRNA derived from EVs collected in MPG membrane could be collected with high efficiency.

The nucleotides [nt] and the concentration of total RNA from UC, MB, PR, and AGC device were measured using a 2100 Bioanalyzer. The amounts of total RNA in PR and in the AGC device was much greater in comparison to the other two methods, because UC and MB only had small RNA of approximately 25 nucleotides; however, PR and the AGC device had both small RNA and longer RNA with 200 to 500 nucleotides (Fig. 4d,e).

To evaluate the number of miRNAs extracted from EVs collected from human serum, a miRNA array analysis was performed (miRNA expression number: 2565 types). The numbers of detected miRNAs were as follows: UC, 354 types; MB, 355 types; PR, 226 types; and the AGC device, 435 types. Thus, among these methods, the AGC device extracted the greatest number of miRNAs (Fig. 4f). A Venn diagram demonstrated the overlap of the number of detected miRNAs between the UC method (as a standard method) and the other three methods. The AGC device had greatest number of types of miRNA in common with the UC method (294 types) in comparison to MB (263 types) and PR (175 types) methods. Moreover, to compare the miRNA expression between the UC method and the other three types of methods (MB, PR, and AGC device), the signal intensity of each detected miRNA was globally normalized. A scatterplot of globally normalized intensities ≥ 20 was prepared. The scatterplot revealed that the miRNA expression level in the AGC device was much higher than that detected with the UC method or other commercially available kits (Fig. 4g–i). These data suggested that AGC device could collect EVs having size and feature distribution with high efficiency among the UC, MB and PR method.

### The miRNA array analysis of EVs collected from human urine

**Figure 5:**
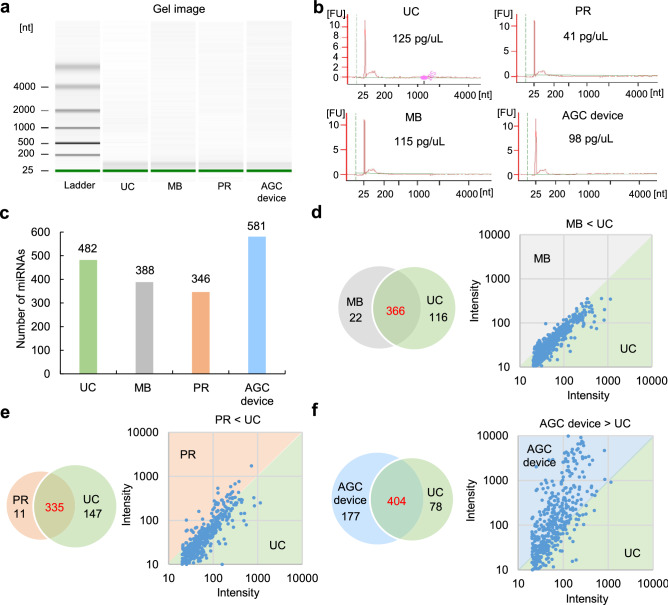
The miRNA array analysis of EVs collected from human urine. (**a**) A gel image of nucleotides [nt] of total RNA extracted from EVs collected from human urine using four EV collection methods (UC, MB, PR, and the AGC device) measured by a 2100 Bioanalyzer. (**b**) The concentration of total RNA extracted from EVs collected from human urine using four EV collection methods (UC, MB, PR, and the AGC device) measured by a 2100 Bioanalyzer. (**c**) The number of miRNAs extracted from EVs collected from human serum using four EV collection methods (UC, MB, PR, and the AGC device). (**d**) A venn diagram of the overlap of the number of detected miRNAs between UC (as the standard method) and the other three methods (MB [**d**], PR [**e**] and the AGC device [**f**]).

A schematic diagram of the miRNA array analysis of EVs collected from human urine is shown in Fig. 5a. The amount of each total RNA from UC, PR, MB, and the AGC device was measured using a 2100 Bioanalyzer. The amount of total RNA in the AGC device was the same level as in other two UC and MB methods, and more than in the PR method (Fig. 5b).

To evaluate the number of miRNAs extracted from EVs collected from human urine, we performed a miRNA array analysis (miRNA expression number: 2565 types). The numbers of detected miRNAs were as follows: UC, 482 types; MB, 388 types; PR, 346 types; and AGC device, 581 types. Thus, in the case of human serum, the AGC device collected the greatest number of miRNAs among these methods (Fig. 5c). A Venn diagram demonstrated the overlap of the number of detected miRNAs between the UC method (as standard method) and the other three types of methods. The AGC device had largest number of miRNA types in common with the UC method (404 types) in comparison to the MB (366 types) and PR (335 types) methods. Moreover, a scatterplot revealed that the miRNA expression level in the AGC device was much higher than that with the UC method and other commercially available kits (Fig. 5d–f). These data suggested that the AGC device was useful for EV collection and the extraction of miRNA from EVs in human serum and urine.

## Discussion

It has recently been reported that the miRNA extracted from EVs plays an important role in the diagnosis and prognosis (recurrence and metastasis) of serious illnesses including tumor disease^32–34^. Consequently, various methods for the collection and isolation of EVs have been developed. However, there are few methods that can simply, quickly and efficiently collect EVs and isolate miRNA using general equipment such as normal centrifuge. We developed an advanced glass membrane column (AGC) device with a size-controlled macro-porous glass (MPG) membrane to overcome these limitations of conventional EV collection and the extraction of miRNA from the EVs. The MPG membrane was composed of SiO_2_ and had a stable co-continuous structure formed by spinodal decomposition. The pore-size of the MPG membrane could be accurately controlled by the heating temperature and the time under the condition of 60% porosity. The PEG coating of the surface significantly inhibited the prehension and adhesion of IgG and DNA molecules to the MPG membrane.

The AGC device was originally designed to be applied to the diagnosis of hepatic tumors using EVs, so the EVs derived from HepG2 cells were used in order to decide the pore size and thickness of MPG membrane. The suitable thickness of the MPG membrane in the AGC device was 1.0 mm, because MPG membranes thinner than 1.0 mm broke easily and showed a decreased capture rate. The suitable pore-size of the MPG in the AGC device was 600 nm, because the capture rate of HepG2-EVs was > 90% and the time required for centrifugation for the AGC device was shorter than 5 min. In contrast, when MPG membranes with a pore size smaller than 600 nm were used, it took more than 30 min to complete the centrifugation of the AGC device. In addition, when the MPG membranes with a smaller pore size were used, the centrifugation of serum samples was not completed, even after more than 1 h. Thus, we used an MPG membrane with a pore-size of 600 nm and a thickness of 1.0 mm in the AGC device. Of course, the pore-size of the MPG membrane can be adjusted to the size of different types of EVs.

In this study, EVs smaller than 600 nm derived from cell culture supernatant, serum and urine were captured with an MPG membrane with a pore size of 600 nm of pore-size. This finding is considered to be dependent on the physical adsorption due to the stable macro-porous co-continuous structure formed by spinodal decomposition in the MPG membrane. This effect is explained by the “Ferry-Renkin equation” proposed by P. J. Ferry and E.M. Renkin. The Ferry-Renkin equation shows that smaller size of solutes than pore size of microporous microfiltration membranes cannot completely pass through the membrane and are captured in the membranes^35–38^. The capture rate of EVs was therefore assumed to be influenced by the size of the macro-pores, the thickness of the MPG membrane, the centrifugation speed of the AGC device and the sample volume. In fact, the capture rates with a larger macro-pore size or a thinner MPG membrane were confirmed to be decreased. The capture rate of an MPG membrane with a pore-size of 3000 nm (thickness: 1.0 mm) was lower < 50% (Fig. S5, Table S1). The centrifugation speed and sample volume had little influence on the capture rate within the limits of normal centrifugation (< 10,000 × *g*) and the AGC device volume (< 500 µL).

There have been a few reports about the application of macro- or meso-porous materials in the accumulation of functional molecules by physisorption^39–42^. Pavel et al. showed the effect of the meso-macro size of hierarchical porous silica on the adsorption and activity of immobilized β-galactosidase^39^. Jo et al. showed the structural effect on the electrochemical performance of ordered porous carbon electrodes for Na-ion batteries^40^. However, we found no published data about the application of porous materials to the collection of EVs; thus, this is the first report to apply the co-continuous structure formed by spinodal decomposition in size-controlled macro-porous glass (MPG) membrane to EV collection and the analysis of miRNA. The AGC device could collect EVs of various sizes and with non-specific populations, and extract miRNA from the EVs captured in MPG membrane with high efficiency when combined with a miRNA extracting solution. Thus, the number of miRNAs in the AGC column detected by the miRNA array were larger than those detected by the three major methods. It is expected that AGC device, which is simple to use and has excellent operability will be effective diagnosing serious illness and predicting the prognosis of patients in the clinical setting using EVs from various types of body fluids. Moreover, in the future, the AGC device may contribute to the collection of specific EVs or viruses by antibody specific to a target antigen in the surface of MPG membrane in the future.

## Conclusion

In this study, an advanced glass membrane column (AGC) device incorporating a size-controlled macro-porous glass (MPG) membrane with co-continuous structure was newly developed in order to overcome the limitations of conventional EV collection and miRNA extraction from the EVs. The AGC device with an MPG membrane (thickness: 1.0 mm, pore size: Φ600 nm) could collect EVs simply and quickly (< 10 min) from cell culture supernatant, serum and urine. In addition, this AGC device could extract miRNA from the EVs captured in the MPG membrane with high efficiency using miRNA extraction solution. The number of miRNAs detected by the miRNA array in the AGC device was larger than the numbers collected by three major methods, regardless of the samples. We suggest that AGC device with MPG membrane will have applications in the diagnosis and prognosis of serious illness based on EVs in various types of body fluids.

## Methods

### Study approval

All experiments on human cells and samples (serum and urine) were approved by the Nagoya University Use Committee, and all methods were performed in accordance with the Declaration of Helsinki.

### Materials and cells

SiO_2_, B_2_O_3_, Na_2_CO_3_, and H_2_SO_4_ were purchased from Tokyo Chemical Industry Co., Ltd. (TCI) (Tokyo, Japan). IgG protein (POD-goat anti mouse IgG) was purchase from BIO-RAD Laboratories Ltd. (Hercules, USA). PBS (D-PBS) was purchased from Sigma-Aldrich Japan (Tolyo, Japan). Tween 20 was purchased from Fujifilm (Tokyo, Japan) 3,3′,5,5′-tetramethylbenzidine and TMB peroxidase substrate were purchased from SeraCare Life Sciences, Inc. (formerly KPL: Kirkegaard & Perry Laboratories, Inc.) (Milford, USA). Serum and urine of healthy donor were purchased from BioIVT (New York, USA). ExoQuick and ExoQuick-TC (Exosome Precipitation Solution) were purchased from Systems Bioscience (California, USA). Thw ExoCap CD9 Kit (Magnetic affinity beads) was purchased from MEDICAL & BIOLOGICAL LABORATORIES Co., Ltd (Nagoya, Japan). The miRNeasy Micro Kit was purchased from QIAGEN (Tokyo, Japan). The TaqMan MicroRNA Reverse Transcription Kit, NoLimits 25 bp, 200 bp, 500 bp DNA Fragment, Qubit dsDNA HS Assay Kit, and M-PER Mammalian Protein Extraction Reagent were purchased from Thermo Fisher Scientific (Massachusetts, USA). HepG2 (Hepatocellular carcinoma cell line) was purchased from the ATCC (Manassas, USA). Dulbecco's Modified Eagle Medium (DMEM) was purchased from Japan Life Technologies Corp. (Tokyo, Japan). Exosome-depleted fetal bovine serum was purchased from System Biosciences Inc. (Palo Alto, CA, USA). Standard exosome was purchased from TheoriaScience (Tokyo, Japan). The anti-CD9 (clone 12A12) and anti-CD63 (clone 8A12) antibodies and CD9/CD63 ELISA kit were purchased from Cosmo Bio (Tokyo, Japan).

### Synthesis of the size-controlled macro-porous glass (MPG) membrane

SiO_2_, B_2_O_3_, and Na_2_CO_3_ were mixed and melted uniformly at 1300 °C, and were then heat-treated at each condition for the macro-porous size (550–740 °C, 3–192 h). SiO_2_–B_2_O_3_–Na_2_O-based glass blocks with spinodal decomposition was obtained and was shape-machined into a glass membrane. The glass membrane was immersed in HNO_3_ (1 mol/L) for leaching, and thereafter was cleaned with NaOH (0.1 mol/L) and 60 °C hot water. In this way, a size-controlled macro-porous glass (MPG) membrane made from silicon dioxide (SiO_2_) was obtained (Fig. 1a,b).

### Fabrication of the advanced glass membrane column (AGC) device

MPG membranes were adjusted in size (diameter: 7.0 mm, thickness: 1.0 mm) and were coated with PEG, and then set into an advanced glass membrane column (AGC) device. The AGC device was composed of upper and lower part made from polypropylene. The upper part held an MPG membrane sandwiched in the vertical direction by washers (outer diameter: 7.0 mm, inner diameter: 4.8 mm) made from polypropylene; its function was to separate and isolate EVs from samples. The lower part was used to collect the supernatant of samples such as culture medium, serum, and urine after centrifugation (Fig. 1c,d). The maximum volume of liquid samples that centrifuged by the AGC device at one time was 500 µL.

### Pore size and porosity of the MPG membrane

The surface area (m^2^/g), porosity (%), and pore size distribution (nm) of the MPG membrane were measured by mercury instruction porosimetry (Auto Pore IV 9510, Micromeritics, GA, USA). The peak of pore size distribution measured by mercury intrusion porosimetry was regard as the pore size.

### Adsorption ratio of immunoglobulin G (IgG) and DNA fragment

IgG protein (POD-goat anti mouse IgG, Biorad) was diluted with PBS. MPG membranes with or without a PEG coating were soaked in the protein solution for 60 min at room temperature. The filters were then washed with the wash-solution 3 times. The filters were soaked in 3 mL of substrate solution, which is a mixture of 3,3′,5,5′-tetramethylbenzidine (TMB) and TMB peroxidase substrate, for 7 min. The TMB reaction was stopped by 2 mol/L H_2_SO_4_ (1.5 mL). The absorbance was measured using a plate reader (MTP-810Lab, CORONA ELECTRIC, Ibaraki, Japan) at a wavelength of 450 nm. The adsorption ratio of IgG with the PEG-coating was expressed with reference to the adsorption of IgG without a PEG coating, with the non-coated condition set as 100%.

DNA fragments (25 bp, 200 bp, 500 bp) were suspended in PBS at a concentration of 3000 ng/mL. Each sample was filtered with AGC device of Φ600 nm, then the concentrations of DNA fragments were measured using the Qubit dsDNA HS Assay Kit (Thermo Fisher Scientific) according to the manufacturer’s protocol.

### Cell culture

HepG2 cells were cultured in DMEM supplemented with 10% EVs-depleted fetal bovine serum and 1% penicillin/streptomycin under 5% CO_2_ at 37 °C. Cell-culture supernatant including EVs was collected after 4 days of incubation, and the EVs derived from HepG2 cells were isolated with each method.

### Measurement of EV size distribution

Isolated EVs derived from HepG2 cells after ultracentrifugation were dispersed in 1 mL of PBS, and the EV size distribution was measured with a dynamic light scattering spectrophotometer (ZETASIZER Nano-ZS, Malvern Instruments Limited, Worcestershire, England).

### Transmission electron microscopy (TEM)

The samples were absorbed onto carbon-coated copper grids (400 mesh) and were stained with 2% phosphotungstic acid solution (pH 7.0) for 10 s. The grids were then observed by a transmission electron microscope (JEM-1400 Plus, JEOL Ltd., Tokyo, Japan) at an acceleration voltage of 80 kV. Digital images (3296 × 2472 pixels) were obtained with a CCD camera (EM-14830RUBY2, JEOL Ltd., Tokyo, Japan).

### EV collection by AGC device

Various types of liquid samples that included EVs, such as cell culture supernatant, serum and urine were filtered through a 0.22 µm filter (Merck KgaA) in the vessel. The filtered samples were placed in the upper part of the AGC device. The AGC device was centrifuged at 6,000 × g for 5 min. The AGC device could be filtrated to 500 µL of sample in each filtration treatment.

### The evaluation of filtration capability by fluorescence measurement

EVs were labeled with 4 µL of green fluorescent PKH67 membrane dye in 1 mL of Diluent C at room temperature for 15 min. The reaction was stopped with 1% BSA solution (1 mL), and the labeled EVs were washed with PBS by centrifugation at 110,000 × g for 80 min. The resulting pellet was resuspended in filtered PBS. The fluorescence intensity of the sample solution was measured with a plate reader spectrophotometer (TECAN infinite M200 PRO, Tecan Japan Co., Ltd., Kanagawa, Japan). The samples were filtered with an AGC device, then the green fluorescence intensity of the supernatant was measured. Fluorescence images of the porous glass surface were observed under a fluorescence microscope (KEYENCE BZ-X710, KEYENCE, Osaka, Japan).

### Scanning electron microscopy (SEM)

EVs captured in the MPG membrane were prepared by fixation with 2% glutaraldehyde buffered in PBS for 90 min and post-fixed in 1.5% osmium tetroxide for 60 min. Samples were next dehydrated in graded ethanol concentrations and then in graded t-Butyl alcohol. They were freeze-dried and coated with platinum using plasma chemical vapor deposition (CVD), and then observed using a field emission scanning electron microscopy (SEM) system (JSM-6301F, JEOL, Tokyo, Japan).

### Conventional methods for EV collection

#### Ultracentrifugation

Collected samples were centrifuged at 3,000 × *g* for 15 min to remove dead cells and cell debris. The supernatant was filtered through a 0.22 µm filter (Merck KGaA) in the vessel. The filtered supernatant was transferred to polycarbonate ultracentrifuge tubes, and centrifuged at 110,000 × *g* for 80 min in a rotor. The supernatant was removed, and then the precipitate was suspended in filtered PBS. The sample was recentrifuged at 110,000 × *g* for 80 min, and the resulting pellet was resuspended in filtered PBS and stored at 4 °C.

#### ExoQuick

Collected samples were centrifuged at 3,000 × *g* for 15 min to remove dead cells and cellular debris. The supernatant was collected, and incubated with ExoQuick at 4 °C for more than 12 h. The sample was centrifuged at 1,500 × *g* for 30 min, and the supernatant was completely removed. The resulting pellet was resuspended in filtered PBS and stored at 4 °C.

#### ExoCap

ExoCap beads were shaken vigorously, and 0.5 mL of ExoCap beads was transferred to a new microtube. The tube was placed in a magnetic stand for 1 min, the supernatant was removed. One milliliter of washing/dilution buffer was added to the collected sample, and the pellet was discarded after centrifugation three times (1; 300 × *g*, 10 min, 2; 2,000 × *g*, 20 min, 3; 10,000 × *g*, 30 min). The supernatant was filtered through a 0.22 µm filter in the vessel and added to purified ExoCap beads, and then mixed slowly for 24 h at room temperature. The sample was table-top centrifuged, and the pellet was collected using a magnetic stand. The collected pellet was washed twice with 1 mL of washing/dilution buffer. The pellet was resuspended in 1 mL of washing/dilution buffer, and the supernatant was carefully removed using a magnetic stand. The resulting pellet was resuspended in filtered PBS and stored at 4 °C.

### RNA extraction and qPCR

Total RNA was extracted from EVs collected from each sample with each method using an miRNeasy Micro Kit. cDNA was synthesized from extracted RNA by a TaqMan MicroRNA Reverse Transcription Kit. The RT conditions were as follows: 1 hold at 37 °C for 1 min, 1 hold at 42 °C for 30 min, and 1 hold at 85 °C for 5 min. The real-time PCR was performed using TaqMan Fast Advanced Master Mix and hsa-miR-21 and primers. Cel-miR-39 was used as reference gene for qPCR calibration. The real-time PCR conditions were as follows: 1 hold at 50 °C for 2 min, 1 hold at 95 °C for 20 s, 40 cycles of 95 °C for 3 s, and 40 cycles of 60 °C for 30 s.

### Western blotting

EV samples captured in AGC device were lysed by adding a M-PER reagent and the device was then centrifuged at 3,000 × g for 5 min. The EV marker proteins of lysed EVs were detected via Western blotting using anti-CD9 antibodies. A commercial EVs (54 ng) isolated from HCT116 using ultracentrifugation was added as a positive control.

### ELISA

CD9/CD63 ELISA kit was used to detect the surface expression of EV markers. 100 µL of Pre-filtered urine treated with 0.22 µm was added to AGC device. The capture rate was evaluated with the concentrations before and after AGC device treatment.

### MiRNA array analysis

The size of extracted RNA was measured by capillary electrophoresis using a 2100 Bioanalyzer (Agilent Technologies, Santa Clara, CA, USA). The obtained miRNA was analyzed using a miRNA array tip (3D-Gene, TORAY, Tokyo, Japan). These data were obtained in cooperation with TORAY Industries, Inc.

### MiRNA extraction from EVs in serum and urine using ultracentrifugation and commercially available kits

Commercially available serum and urine were centrifuged (15 min, 4 °C, 3,000 × *g*) to remove apoptotic bodies, and then the samples were centrifuged (15 min, 4 °C, 12,000 × *g*) to remove cellular debris before use. These samples were ultracentrifuged (2 h, 4 °C, 110,000 × *g*), and then the supernatant was then discarded. PBS (0.5 mL) was added to the collected EVs, and the EVs were suspended in PBS. Serum and urine were filtered with a 0.22 µm filter before use. The EVs were collected from 0.5 mL of serum and urine samples according to the instruction of the respective kit manufacturer.

### Statistical analysis of cell properties

The numerical values are presented as the mean ± standard error. Each experiment was repeated three times. Statistical significance was evaluated using an unpaired Student’s *t*-test for comparisons between two groups. *P*-values of < 0.05 were considered to indicate statistical significance. All of the statistical analyses were performed using the SPSS software package.

## Supplementary Information

Supplementary Information.

## References

[1] Fan Z, Yu J, Lin J, Liu Y, Liao Y (2019). Exosome-specific tumor diagnosis via biomedical analysis of exosome-containing microRNA biomarkers. Analyst.

[2] Sindeeva O (2019). New frontiers in diagnosis and therapy of circulating tumor markers in cerebrospinal fluid in vitro and in vivo. Cells.

[3] LeBleu VS, Kalluri R (2020). Exosomes as a multicomponent biomarker platform in cancer. Trends Cancer.

[4] Shin H (2020). Early-stage lung cancer diagnosis by deep learning-based spectroscopic analysis of circulating exosomes. ACS Nano.

[5] Huang M (2020). Homogeneous, low-volume, efficient, and sensitive quantitation of circulating exosomal PD-L1 for cancer diagnosis and immunotherapy response prediction. Angew. Chem. Int. Ed. Engl..

[6] Dong S (2020). Beehive-inspired macroporous SERS probe for cancer detection through capturing and analyzing exosomes in plasma. ACS Appl. Mater. Interfaces..

[7] Théry C (2015). Cancer: Diagnosis by extracellular vesicles. Nature.

[8] Lee J (2019). Enhanced paper-based ELISA for simultaneous EVs/exosome isolation and detection using streptavidin agarose-based immobilization. Analyst.

[9] Liu C (2017). Field-free isolation of exosomes from extracellular vesicles by microfluidic viscoelastic flows. ACS Nano.

[10] Hildonen S, Skarpen E, Halvorsen TG, Reubsaet L (2016). Isolation and mass spectrometry analysis of urinary extraexosomal proteins. Sci. Rep..

[11] Xu H, Liao C, Zuo P, Liu Z, Ye BC (2018). Magnetic-based microfluidic device for on-chip isolation and detection of tumor-derived exosomes. Anal. Chem..

[12] Singhto N, Vinaiphat A, Thongboonkerd V (2019). Discrimination of urinary exosomes from microvesicles by lipidomics using thin layer liquid chromatography (TLC) coupled with MALDI-TOF mass spectrometry. Sci. Rep..

[13] Zhang Y (2017). Hypothalamic stem cells control ageing speed partly through exosomal miRNAs. Nature.

[14] Zhao L (2017). Isolation and Identification of miRNAs in exosomes derived from serum of colon cancer patients. J. Cancer.

[15] Liang LG (2017). An integrated double-filtration microfluidic device for isolation, enrichment and quantification of urinary extracellular vesicles for detection of bladder cancer. Sci. Rep..

[16] Gheinani AH (2018). Improved isolation strategies to increase the yield and purity of human urinary exosomes for biomarker discovery. Sci. Rep..

[17] Morad G (2019). Tumor-derived extracellular vesicles breach the intact blood-brain barrier via transcytosis. ACS Nano.

[18] Yukawa H (2018). Imaging of angiogenesis of human umbilical vein endothelial cells by uptake of exosomes secreted from hepatocellular carcinoma cells. Sci. Rep..

[19] Liu F (2017). The exosome total isolation chip. ACS Nano.

[20] Shi L (2019). Rapid and label-free isolation of small extracellular vesicles from biofluids utilizing a novel insulator based dielectrophoretic device. Lab. Chip.

[21] Luo X, An M, Cuneo KC, Lubman DM, Li L (2018). High-performance chemical isotope labeling liquid chromatography mass spectrometry for exosome metabolomics. Anal. Chem..

[22] Chen J, Xu Y, Lu Y, Xing W (2018). Isolation and visible detection of tumor-derived exosomes from plasma. Anal. Chem..

[23] Weng Y (2016). Effective isolation of exosomes with polyethylene glycol from cell culture supernatant for in-depth proteome profiling. Analyst.

[24] Fang X (2019). Highly efficient exosome isolation and protein analysis by an integrated nanomaterial-based platform. Anal. Chem..

[25] Yasui T (2017). Unveiling massive numbers of cancer-related urinary-microRNA candidates via nanowires. Sci. Adv..

[26] Liu C (2019). λ-DNA- and aptamer-mediated sorting and analysis of extracellular vesicles. J. Am. Chem. Soc..

[27] Zhao J (2020). Thermophoretic detection of exosomal microRNAs by nanoflares. J. Am. Chem. Soc..

[28] Seward TP, Uhlman DR, Turnbull D (1968). Phase separation in the system BaO-SiO_2_. J. Am. Ceram. Soc..

[29] Haller W, Blackburn DH, Wagstaff FE, Charles RJ (1970). Metastable immiscibility surface in the system Na_2_O-B_2_O_3_-SiO_2_. J. Am. Ceram. Soc..

[30] Elmer TH, Nordberg ME, Carrier GB, Korda EJ (1970). Phase separation in borosilicate glasses as seen by electron microscopy and scanning electron microscopy. J. Am. Ceram. Soc..

[31] Haller W, Blackburn DH, Simmons JH (1974). Miscibility gaps in alkali-silicate binaries-data and thermodynamic interpretation. J. Am. Ceram. Soc..

[32] Madeo M (2018). Cancer exosomes induce tumor innervation. Nat. Commun..

[33] Kamerkar S (2017). Exosomes facilitate therapeutic targeting of oncogenic KRAS in pancreatic cancer. Nature.

[34] Zhang L (2015). Microenvironment-induced PTEN loss by exosomal microRNA primes brain metastasis outgrowth. Nature.

[35] Baker RW (2012). Membrane technology and applications.

[36] Ferry JD (1936). Ultrafilter membranes and ultrafiltration. Chem. Rev..

[37] Renkin EM (1955). Filtration, diffusion and molecular sieving through porous cellulose membranes. J. Gen. Physiol..

[38] Baker RW, Strathmann H (1970). Ultrafiltration of macromolecular solutions with high-flux membranes. J. Appl. Polym. Sci..

[39] Pavel IA (2017). Effect of meso vs macro size of hierarchical porous silica on the adsorption and activity of immobilized β-galactosidase. Langmuir.

[40] Jo C, Park Y, Jeong J, Lee KT, Lee J (2015). Structural effect on electrochemical performance of ordered porous carbon electrodes for Na-ion batteries. ACS Appl. Mater. Interfaces.

[41] Kim T, Jung G, Yoo S, Suh KS, Ruoff RS (2013). Activated graphene-based carbons as supercapacitor electrodes with macro- and mesopores. ACS Nano.

[42] Xu E (2019). Magnetic (Zn-St)_10_Fe0_n_ (n = 1, 2, 3, 4) framework of macro-mesoporous biomaterial prepared via green enzymatic reactive extrusion for dye pollutants removal. ACS Appl. Mater Interfaces.

