# Supplementary material for: Co-continuous structural effect of size-controlled macro-porous glass membrane on extracellular vesicle collection for the analysis of miRNA
Source: Sci Rep. 2021 Apr 21;11:8672. doi: 10.1038/s41598-021-87986-2 (PMC8060318; doi:10.1038/s41598-021-87986-2)
Supplement: Supplementary file 1 — Supplementary Information. [file 41598_2021_87986_MOESM1_ESM.pdf]

Supplementary Information

Co-continuous structural effect of size-controlled macro-porous glass membrane on extracellular vesicle collection for the analysis of miRNA

Hiroshi Yukawa [1,2,3,4 *], Shuji Yamazaki [5], Keita Aoki [2], Kengo Muto [2], Naoto Kihara [5], Kazuhide Sato [1,4,6], Daisuke Onoshima [1], Takahiro Ochiya [7], Yasuhito Tanaka [8], and Yoshinobu Baba [1,2,3,9*]

*** Address correspondence to**

Hiroshi Yukawa

Institute of Nano-Life-Systems, Institutes of Innovation for Future Society, Nagoya University, Furo-cho, Chikusa-ku, Nagoya 464-8603, Japan.

Tel.: ++81-52-789-5654; Fax. : ++81-52-789-5177

E-mail: h.yukawa@nanobio.nagoya-u.ac.jp

Yoshinobu Baba

Department of Biomolecular Engineering, Graduate School of Engineering, Nagoya University, Furo-cho, Chikusa-ku, Nagoya 464-8603, Japan.

Tel.: ++81-52-789-4664; Fax. : ++81-52-789-4666

E-mail: babaymtt@chembio.nagoya-u.ac.jp

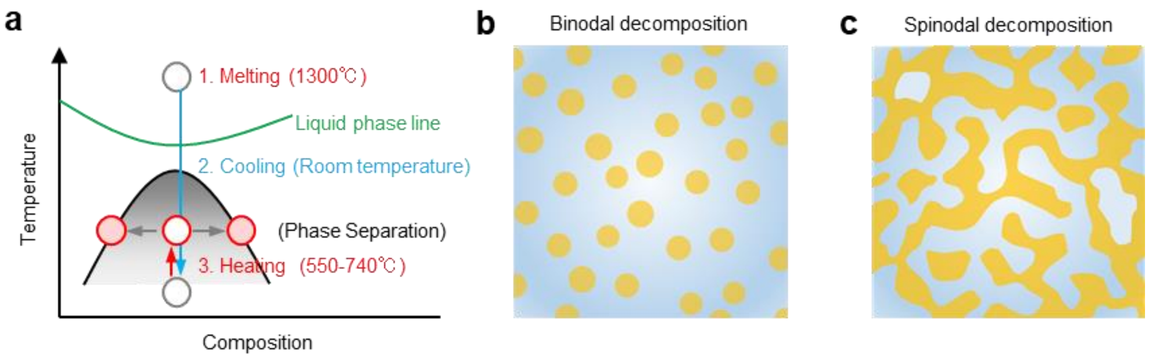

Supplementary Figure 1. Phase diagram (a) and decomposition (b,c) of SiO_2-B_2O_3 Na_2O-based glass.

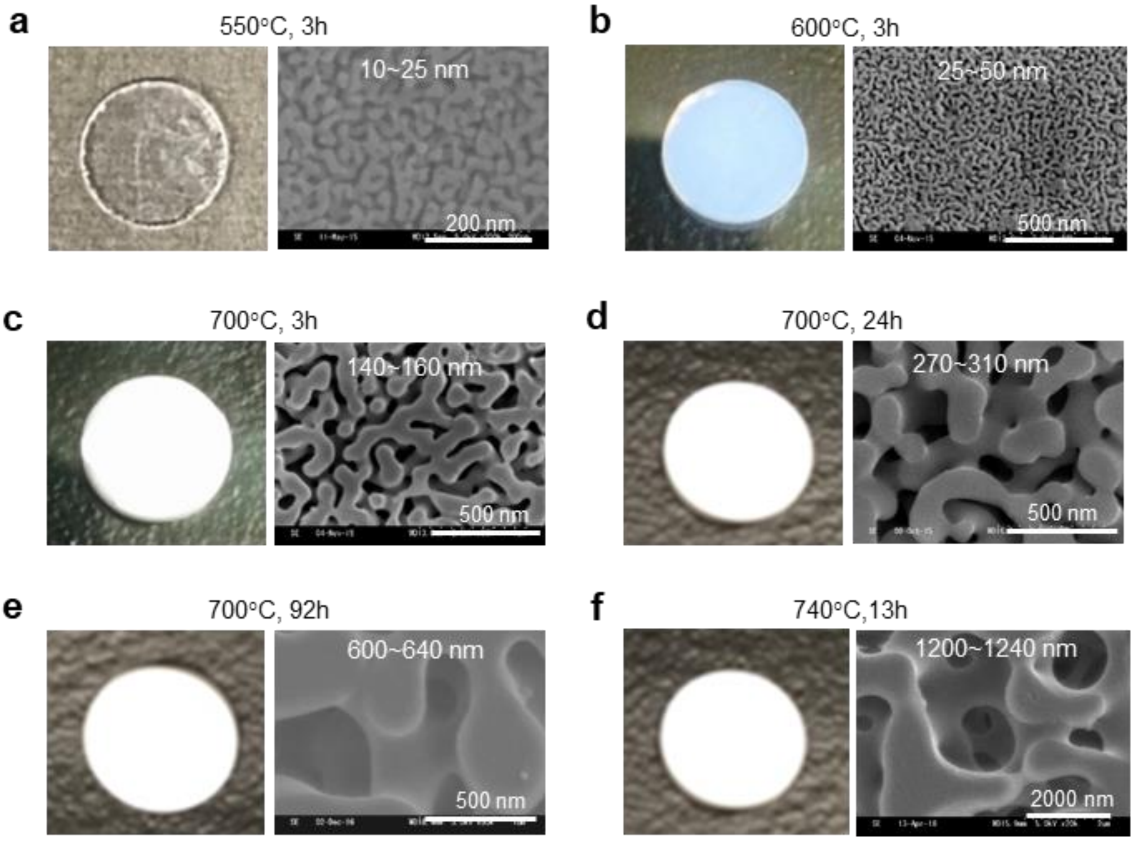

Supplementary Figure 2. Photograph and TEM images of size-controlled macro-porous glass (MPG) membranes; (a) heating temperature: 550°C, heating time: 3 h. (b) 600°C, 3 h. (c) 700°C, 3 h. (d) 700°C, 24 h. (e) 700°C, 92 h. (f) 740°C, 13h

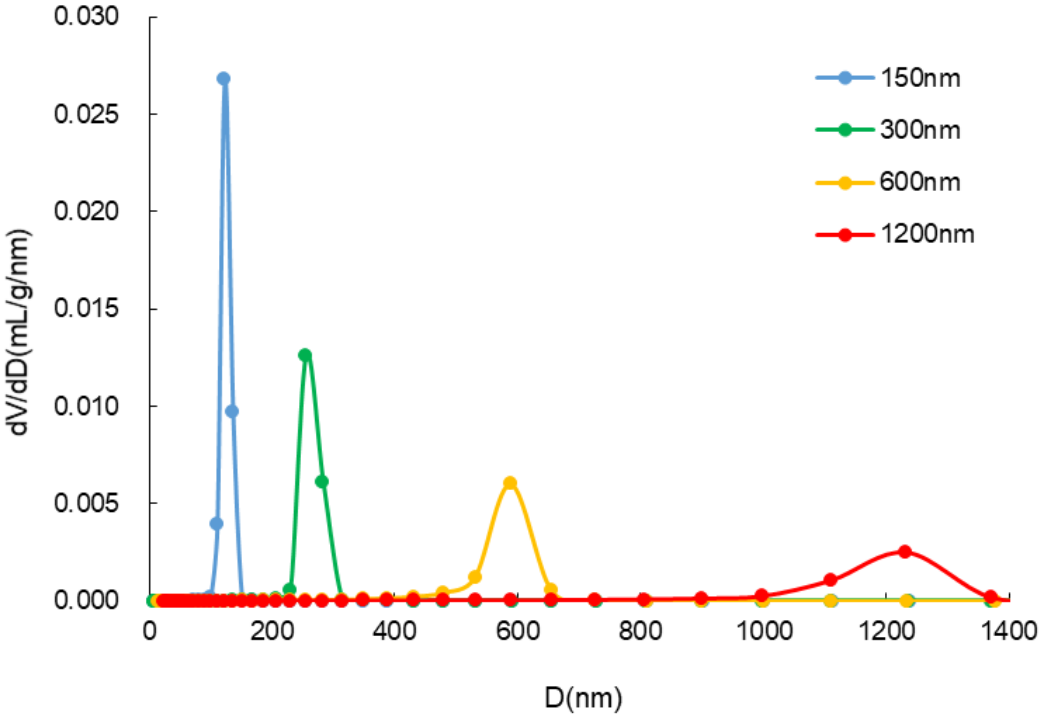

Supplementary Figure 3. Pore size distribution (nm) of MPG membranes (Φ150 nm, Φ300 nm, Φ600 nm, Φ1200 nm shown as Figure S2c~f) by using a mercury instruction porosimetry.

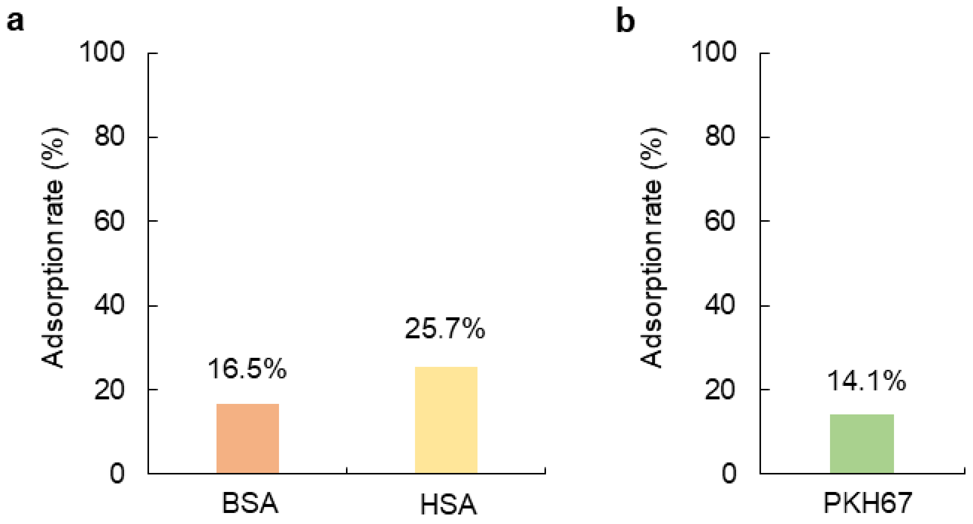

Supplementary Figure 4. The adsorption rate (%) of bovine serum albumin (BSA) and human serum albumin (HAS) (a), and fluorescent regent (PKH67) (b) in a PEG coated MPG membrane (pore-size: Φ600 nm, thickness: 1mm)

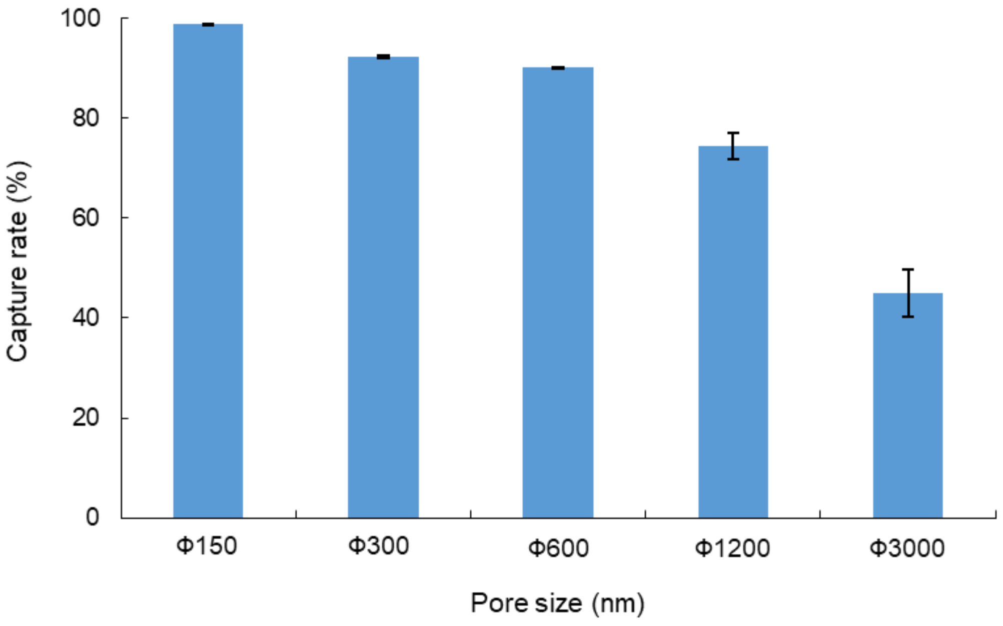

Supplementary Figure 5. The capture rate (%) of HepG2-EVs in PEG coated MPG membranes (pore-size: Φ150 nm, Φ300 nm, Φ600 nm, Φ1200 nm, and Φ3000 nm; thickness: 1 mm).

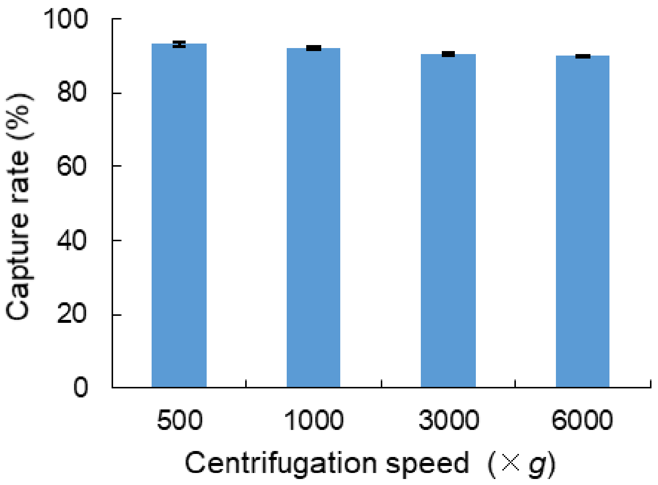

Supplementary Figure 6. The capture rate (%) of HepG2-EVs in a PEG coated MPG membrane (pore-size: Φ600 nm, thickness: 1 mm) by changes of centrifugation speed (500 × g, 1000 × g, 3000 × g, and 6000 × g).

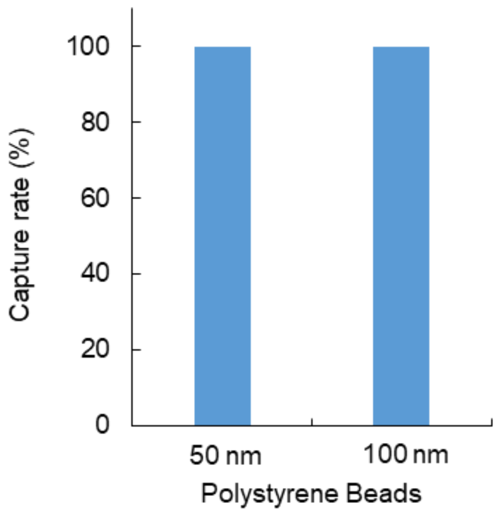

Supplementary Figure 7. The capture rate (%) of polystyrene beads (diameter: 50 nm and 100 nm) in a PEG coated MPG membrane (pore-size: Φ600 nm, thickness: 1mm).

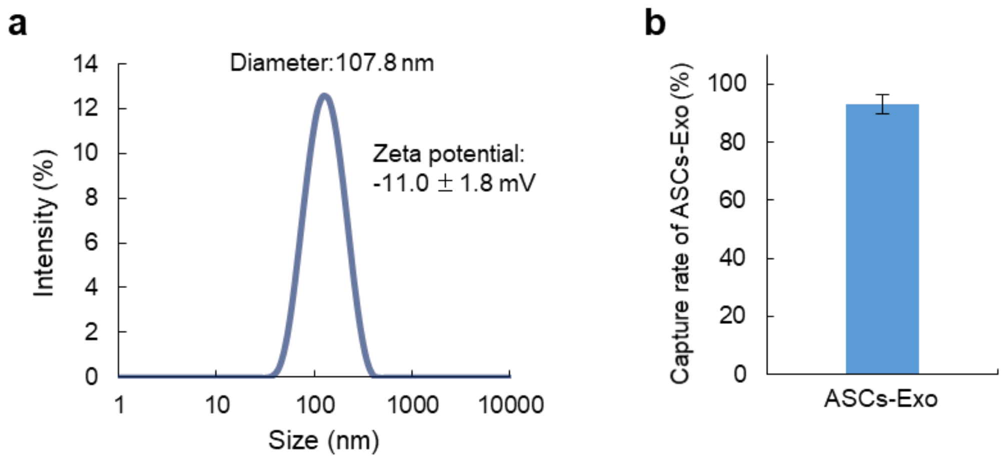

Supplementary Figure 8. (a) The size distribution and zeta potential of EVs collected from the cell culture supernatant of adipose-tissue derived stem cells (ASCs-EVs). (b) The capture rate (%) of ASCs-EVs in a PEG coated MPG membrane (pore-size: Φ600 nm, thickness: 1 mm).

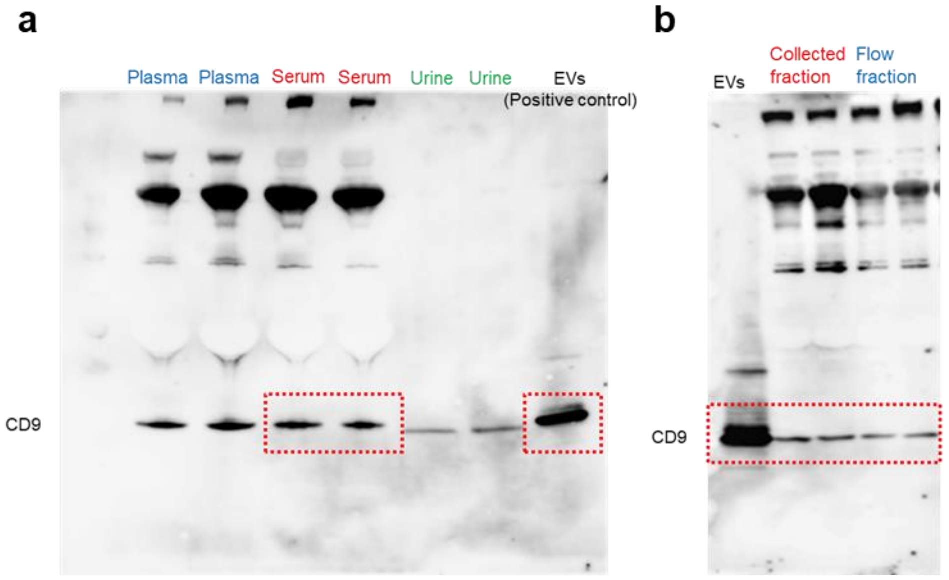

Supplementary Figure 9. (a) The western blotting of CD9 in plasma, serum, and urine showed as full-length gels and blots. (b) The western blotting of CD9 in the collected and flow fraction of serum treated by AGC device showed as full-length gels and blots. The areas represented by red dot lines were shown in main manuscript.

Supplementary Table 1. The time required for centrifugation to collect EVs in the AGC device under various conditions.

		Centrifugation Speed			
	Pore-size	500 × *g*	1000 × *g*	3000 × *g*	6000 × *g*
Thickness 1 mm	Φ150 nm	> 5 min	> 5 min	> 5 min	> 5 min
	Φ300 nm	> 5 min	> 5 min	> 5 min	> 5 min
	Φ600 nm	> 5 min	< 3 min	< 1 min	< 30 s
	Φ1200 nm	< 3 min	< 1 min	< 15 s	< 15 s
		Centrifugation Speed			
	Thickness	500 × *g*	1000 × *g*	3000 × *g*	6000 × *g*
Pore-size Φ600 nm	0.5 mm	< 3 min	< 1 min	< 1 min	< 15 s
	0.8 nm	< 3 min	< 3 min	< 1 min	< 15 s
	1.0 nm	> 5 min	< 3 min	< 1 min	< 30 s
